# Editorial: Metabolic associated fatty liver disease: clinical perspectives from pathogenesis to diagnosis and treatment

**DOI:** 10.3389/fendo.2023.1206642

**Published:** 2023-06-30

**Authors:** Zhe Huang, Dewei Ye, Kerry Loomes, Kenneth Kingyip Cheng, Hannah Xiaoyan Hui

**Affiliations:** ^1^ Department of Genetics and Developmental Science, School of Life Sciences and Biotechnology, Shanghai Jiao Tong University, Shanghai, China; ^2^ Key Laboratory of Metabolic Phenotyping in Model Animals, Guangdong Pharmaceutical University, Guangzhou, China; ^3^ School of Biological Sciences and Maurice Wilkins Centre, University of Auckland, Auckland, New Zealand; ^4^ Department of Health Technology and Informatics, The Hong Kong Polytechnic University, Hong Kong, Hong Kong SAR, China; ^5^ School of Biomedical Sciences, The Chinese University of Hong Kong, Hong Kong, Hong Kong SAR, China

**Keywords:** MAFLD, obesity, biological marker, calcium signalling, metabolic disorders

Obesity is a global epidemic (1, 2) with profound pathological implications for the liver, leading to a spectrum of liver abnormalities now named metabolic-associated fatty liver disease (MAFLD) (3). The “multiple hit” hypothesis proposes that MAFLD arises through multiple insults acting together (4). However, the exact identities of these “hits”, their interplay and dynamic changes, together with the onset and progression of MAFLD are far from clear. Accordingly, it is not surprising that pharmacological treatment agents for MAFLD are still lacking. In addition, diagnosis of MAFLD remains a challenge since no specific diagnostic markers are yet available to achieve non-invasive diagnosis and staging of the disease.

As a key integrator of the body’s energy needs and metabolic homeostasis, the liver receives multiple afferent metabolic signals from metabolically active organs. Within the context of MAFLD pathogenesis, there is increasing research focus on the dysregulated organ crosstalk between the liver and other peripheral organs, including adipose tissue, skeletal muscle, gut, and microbiome (5). In particular, various mechanisms, such as chronic inflammation, endoplasmic reticulum (ER) stress, cellular senescence, insulin resistance, aberrant lipid metabolism, oxidative stress, and mitochondrial dysfunction, are implicated in the development and progression of the disease (6–9). A better understanding of the pathogenic events during the initiation and progression of MAFLD is therefore of great importance to establish more precise and non-invasive diagnostic methods and to inspire novel approaches for the management and prevention of MAFLD.

This Research Topic focuses on how insights into the molecular mechanisms underlying MAFLD are improving our knowledge of diagnostic biomarkers and therapeutic interventions for the disease. This Research Topic contains seven articles: six original research papers and one systematic review.

The actual prevalence of MAFLD is probably underestimated due to a limitation in diagnosis. Liver biopsy remains the gold standard in the diagnosis of MAFLD but suffers from poor patient acceptance due to its invasive nature (10). Therefore, non-invasive and highly-sensitive diagnostic approaches are warranted for early diagnosis of MAFLD. In a cross-sectional study conducted by Duan et al., the authors identify a positive association between cardiometabolic index (CMI) and MAFLD risk. CMI is calculated by multiplying the ratio of triglycerides and high-density lipoprotein cholesterol (TG/HDL-C) by waist-to-height ratio. High CMI shows excellent predictive value for MAFLD. Although MAFLD is strongly associated with obesity, there is nevertheless 8-19% of the population with MAFLD that are non-obese (11). Non-obese individuals with MAFLD have a significantly higher risk of developing cardiovascular diseases (CVDs) than obese individuals with MAFLD (12). Studies on the prediction of MAFLD in the non-obese population are more limited than studies in the obese population. In a longitudinal study analyzed by Li K. et al., the atherogenic index of plasma (AIP), calculated by the logarithm of TG/HDL-C, was identified as a strong independent risk factor for new-onset MAFLD in non-obese individuals, especially in those with low body mass index (BMI). These studies suggest CMI and AIP as potential markers for the diagnosis of MAFLD in obese and non-obese individuals, respectively.

Another retrospective case-control study that included 1,058 Chinese participants was conducted by Liu et al. The correlation between MAFLD and 22 metabolism-related indexes and the predictive values of these 22 indicators were calculated. The authors’ findings revealed that the best predictors of overall MAFLD, obesity, lean diabetes mellitus, and type 2 diabetes mellitus (T2DM) MAFLD subgroups were fatty liver index (FLI), lipid accumulation product (LAP), waist circumference-triglyceride index (WTI), and FLI, respectively. 

MAFLD is not only associated with obesity and metabolic disorders but also strongly correlates with CVDs. From their cross-sectional datasets, Lei et al. found that MAFLD is significantly associated with 12-lead ECG-diagnosed atrial fibrillation. Furthermore, in the retrospective cohort, MAFLD was closely associated with the incidence of atrial fibrillation.

Most patients with MAFLD start from simple steatosis, which is generally a benign condition. Approximately one-third of these patients develop NASH, the advanced form of MAFLD that can prompt collagen synthesis and deposition, leading to cirrhosis and even hepatocellular carcinoma (13). Therefore, understanding the mechanisms that govern the transition from simple steatosis to NASH is critical for the potential prevention of NASH progression. This is the focus of the study by Li X. et al., which analyzed gene expression profiles of liver tissues from patients with MAFLD obtained from various publicly available datasets. The authors identify a downregulation of the metallothionein pathway during the transition from steatosis to NASH. Interestingly, overexpression of metallothionein 1M *in vitro* protects hepatocytes from palmitate-induced lipotoxicity suggesting metallothionein as a potential intervention target for MAFLD progression.

The Ca^2+^ signaling pathway, especially abnormal Ca^2+^ transport within the mitochondria-associated endoplasmic reticulum membranes (MAM) region, is closely associated with cellular activities such as lipid synthesis and transportation, regulation of mitochondrial dynamics, inflammatory vesicle formation, ERS, autophagy and apoptosis (14, 15). Furthermore, Ca^2+^ signaling within the MAM region is implicated in hepatic gluconeogenesis, adipogenesis, inflammation, and other biological processes (16). Tang et al. interrogated the potential role of Ca^2+^ signaling within the MAM region in non-alcoholic fatty liver disease (NAFLD) pathogenesis. To this end, a case-control study was conducted in which 2,701 subjects from the Chinese Han population were enrolled and genotyped for six genetic variants of the Ca^2+^ transport-associated genes, *HSPA5* and *ITPR2*, and the impact of these variants on NAFLD risk was assessed. The study identified a correlation between NAFLD risk and the variant genotypes of *HSPA5* (rs12009 and rs430397) and *ITPR2* (rs11048570), thereby implicating Ca^2+^ signaling in NAFLD pathogenesis.

In the review article by Wang et al., a meta-analysis of randomized controlled trials (RCTs) was conducted to assess the efficacy of probiotics in the treatment of MAFLD, mainly in terms of liver function, glucose and lipid metabolism, and inflammation. This meta-analysis included 772 patients from 15 studies and found that the probiotic therapies efficiently decreased concentrations of liver injury markers and insulin resistance in patients with MAFLD versus control individuals. These findings indicate that probiotic supplementation can decrease liver enzyme concentrations and beneficially regulate glucose metabolism in patients with MAFLD. Subsequent analyses revealed that age, baseline body mass index, and the duration of intervention, may influence the outcomes of the probiotic therapy.

## Author contributions

All authors listed have made a substantial, direct, and intellectual contribution to the work and approved it for publication.

## References

[1] Allison DB Downey M Atkinson RL Billington CJ Bray GA Eckel RH. Obesity as a disease: a white paper on evidence and arguments commissioned by the council of the obesity society. Obesity (2008) 16:1161–77. doi: 10.1038/oby.2008.231 18464753

[2] Crewe C An YA Scherer PE. The ominous triad of adipose tissue dysfunction: inflammation, fibrosis, and impaired angiogenesis. J Clin Invest (2017) 127:74–82. doi: 10.1172/JCI88883 28045400 PMC5199684

[3] Eslam M Newsome PN Sarin SK Anstee QM Targher G Romero-Gomez M. A new definition for metabolic dysfunction-associated fatty liver disease: an international expert consensus statement. J Hepatol (2020) 73:202–9. doi: 10.1016/j.jhep.2020.03.039 32278004

[4] Buzzetti E Pinzani M Tsochatzis EA. The multiple-hit pathogenesis of non-alcoholic fatty liver disease (NAFLD). Metabolism (2016) 65:1038–48. doi: 10.1016/j.metabol.2015.12.012 26823198

[5] Zhang X Ji X Wang Q Li JZ. New insight into inter-organ crosstalk contributing to the pathogenesis of non-alcoholic fatty liver disease (NAFLD). Protein Cell (2018) 9:164–77. doi: 10.1007/s13238-017-0436-0 PMC5818366 28643267

[6] Lebeaupin C Vallee D Hazari Y Hetz C Chevet E Bailly-Maitre B. Endoplasmic reticulum stress signalling and the pathogenesis of non-alcoholic fatty liver disease. J Hepatol (2018) 69:927–47. doi: 10.1016/j.jhep.2018.06.008 29940269

[7] Carotti S Aquilano K Valentini F Ruggiero S Alletto F Morini S. An overview of deregulated lipid metabolism in nonalcoholic fatty liver disease with special focus on lysosomal acid lipase. Am J Physiol Gastrointest Liver Physiol (2020) 319:G469–80. doi: 10.1152/ajpgi.00049.2020 32812776

[8] Ogrodnik M Miwa S Tchkonia T Tiniakos D Wilson CL Lahat A. Cellular senescence drives age-dependent hepatic steatosis. Nat Commun (2017) 8:15691. doi: 10.1038/ncomms15691 28608850 PMC5474745

[9] Lin H Wang L Liu Z Long K Kong M Ye D. Hepatic MDM2 causes metabolic associated fatty liver disease by blocking triglyceride-VLDL secretion *via* ApoB degradation. Adv Sci (Weinh) (2022) 9:e2200742. doi: 10.1002/advs.202200742 35524581 PMC9284139

[10] Ando Y Jou JH. Nonalcoholic fatty liver disease and recent guideline updates. Clin Liver Dis (Hoboken) (2021) 17:23–8. doi: 10.1002/cld.1045 PMC7849298 33552482

[11] Drew L. Fighting the fatty liver. Nature (2017) 550:S102–s103. doi: 10.1038/550S102a 29019968

[12] Yoshitaka H Hamaguchi M Kojima T Fukuda T Ohbora A Fukui M. Nonoverweight nonalcoholic fatty liver disease and incident cardiovascular disease: a *post hoc* analysis of a cohort study. Med (Baltimore) (2017) 96:e6712. doi: 10.1097/MD.0000000000006712 PMC5419911 28471965

[13] Diehl AM Day C. Cause, pathogenesis, and treatment of nonalcoholic steatohepatitis. N Engl J Med (2017) 377:2063–72. doi: 10.1056/NEJMra1503519 29166236

[14] Filadi R Theurey P Pizzo P. The endoplasmic reticulum-mitochondria coupling in health and disease: molecules, functions and significance. Cell Calcium (2017) 62:1–15. doi: 10.1016/j.ceca.2017.01.003 28108029

[15] Hayashi T Rizzuto R Hajnoczky G Su TP. MAM: more than just a housekeeper. Trends Cell Biol (2009) 19:81–8. doi: 10.1016/j.tcb.2008.12.002 PMC2750097 19144519

[16] Arruda AP Hotamisligil GS. Calcium homeostasis and organelle function in the pathogenesis of obesity and diabetes. Cell Metab (2015) 22:381–97. doi: 10.1016/j.cmet.2015.06.010 PMC4558313 26190652

